# Keeping the right time in space: importance of circadian clock and sleep for physiology and performance of astronauts

**DOI:** 10.1186/2054-9369-1-23

**Published:** 2014-10-21

**Authors:** Jin-Hu Guo, Wei-Min Qu, Shan-Guang Chen, Xiao-Ping Chen, Ke Lv, Zhi-Li Huang, Yi-Lan Wu

**Affiliations:** Key Laboratory of Gene Engineering of the Ministry of Education, State Key Laboratory of Biocontrol, School of Life Sciences, Sun Yat-sen University, Guangzhou, 510006 China; Department of Pharmacology, School of Basic Medical Sciences, Fudan University, Shanghai, 200032 China; National Key Laboratory of Human Factors Engineering, China Astronaut Research and Training Center, Beijing, 100094 China; State Key Laboratory of Space Medicine Fundamentals and Application, China Astronaut Research and Training Center, Beijing, 100094 China

**Keywords:** Space, Circadian rhythm, Sleep, Performance, Countermeasure

## Abstract

The circadian clock and sleep are essential for human physiology and behavior; deregulation of circadian rhythms impairs health and performance. Circadian clocks and sleep evolved to adapt to Earth’s environment, which is characterized by a 24-hour light–dark cycle. Changes in gravity load, lighting and work schedules during spaceflight missions can impact circadian clocks and disrupt sleep, in turn jeopardizing the mood, cognition and performance of orbiting astronauts. In this review, we summarize our understanding of both the influence of the space environment on the circadian timing system and sleep and the impact of these changes on astronaut physiology and performance.

The circadian clock and sleep, two dynamically associated physiological systems, are fundamental for physiology and contribute to optimal behavior and performance [1]. High performance efficacy is critical for astronauts to accomplish tasks on space missions. During missions, astronauts are exposed to the space environment, which is dramatically different from that on Earth [1, 2]. The circadian clock and sleep are subject to change in the space environment, which further alters physiology and performance. Understanding the influence of the space environment on the circadian clock, sleep and performance is highly important for the management of space travel and will be of critical importance for future long-duration space exploration.

## Circadian clock and sleep

The circadian clock controls nearly all patterns of human biology, including brain-wave activity, sleep-wake cycles, body temperature, hormone secretion, blood pressure, cell regeneration, metabolism and behavior, which display ~24-hour periodicity [3]. In addition, cognition and performance are also under circadian control [4].

In mammals and humans, the circadian clock consists of a central clock and peripheral tissue clocks. The central clock is located in the suprachiasmatic nuclei (SCN) of the hypothalamus, which functions as the master pacemaker by synchronizing physiological rhythms in accordance with Earth’s cycling environment. The central clock operates to synchronize the clocks in peripheral tissues [3, 5].

At the molecular level, mammalian clocks are composed of positive and negative elements that drive the rhythmicity of gene expression. BMAL1 and CLOCK are two positive elements that bind to the promoters of the *Period* (*Per1* and *Per2*) genes and *Cryptochrome* (*Cry1 and Cry2)* genes and facilitate their transcription. The protein products PER and CRY accumulate, dimerize and act as negative elements that bind to positive elements, which leads to repression of their own transcription. These positive and negative circuits form a negative feedback loop that is essential to eukaryotic circadian clocks [5].

The free-running period of the human clock is slightly longer than 24 hours, but it is entrained to be 24 hours by synchronization to the daily cycling environmental factors. These factors, such as light and temperature, act as zeitgebers (external time-giving cues) that set the phase of circadian rhythms [6–8]. Among the zeitgebers, light is the major input into the central clock, which coordinates internal physiology with the external environment to optimize survival [9]. Bright light >2,500 lux is sufficient to entrain the human circadian clock, and light is often used in the treatment of disorders associated with circadian desynchronization [10].

Sleep is a large component of the daily circadian cycle and is cooperatively regulated by homeostatic and circadian factors. Normal waking is associated with neuronal activity in several chemically defined ascending arousal systems [11]. Ascending arousal systems include monoaminergic neurons in the brainstem and posterior hypothalamus, cholinergic neurons in the brainstem and basal forebrain, and orexin neurons in the lateral hypothalamus. An important set of sleep-related neurons are located in the preoptic hypothalamus, including the ventrolateral preoptic (VLPO) area and the median preoptic nucleus [11].

Neurons in each of the monoaminergic nuclei fire more rapidly during wakefulness than during sleep; firing slows significantly during non-rapid eye movement (non-REM, or NREM) sleep and stops altogether during REM sleep [5, 12, 13]. Orexin neurons are similarly more active during wakefulness than during sleep [14]. Many basal forebrain neurons, including most cholinergic neurons, are active during both wakefulness and REM sleep [5]. VLPO neurons are primarily active during sleep and contain the inhibitory neurotransmitters galanin and GABA [15, 16]. The VLPO area inhibits the ascending arousal regions and is in turn inhibited by them, thus forming a mutually inhibitory system resembling what electrical engineers call a “flip-flop switch” [17, 18].

**Figure 1:**
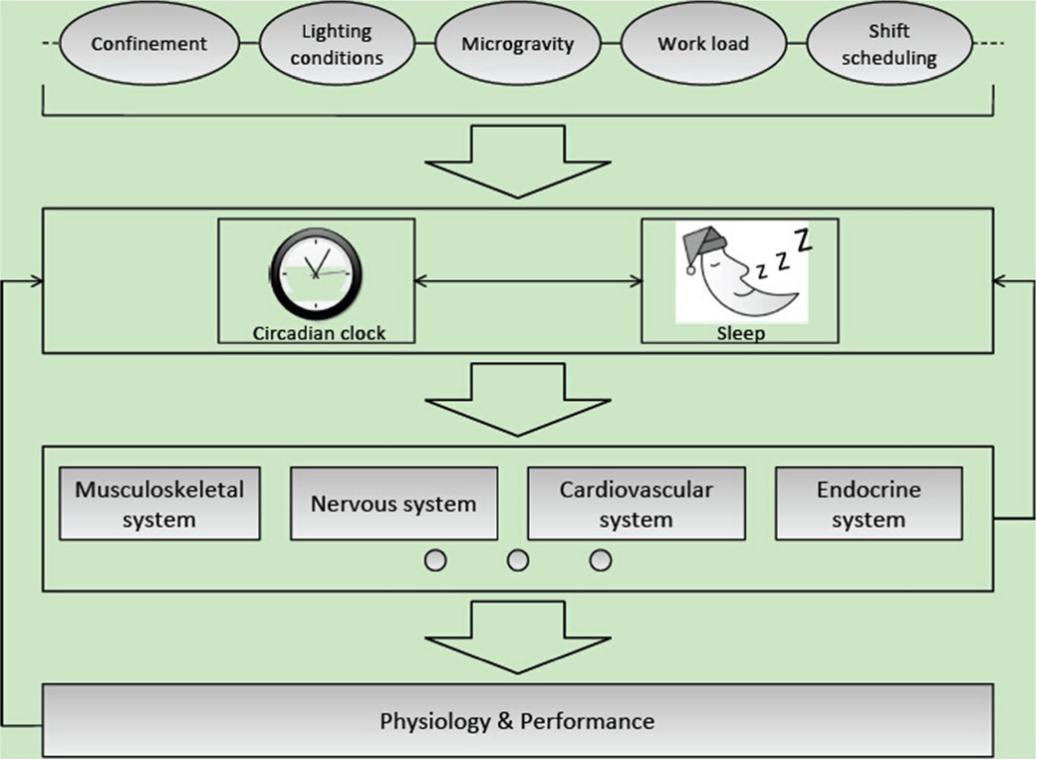
**Network diagram of the circadian clock, sleep, physiology and behavior in space.** In space, a variety of environmental factors are involved in the regulation of the circadian clock and sleep. The circadian clock and sleep regulate each other. The alignment of the circadian clock and sleep is critical for physiology, behavior and performance. In turn, behavior and performance can feed back to affect the circadian clock and sleep.

The circadian propensity for sleep increases during the sleep state, thus ensuring continued sleep despite the diminishing homeostatic need for it toward the end of the sleep cycle. Anatomical and functional evidence indicates that there is a relationship between the SCN and the sleep-wake system. The SCN has relatively modest projections into the VLPO and orexin neurons [19–21]. However, the major output is directed toward the adjacent subparaventricular zone and the dorsomedial nucleus of the hypothalamus. Cell-specific lesions in the ventral subparaventricular zone or the dorsomedial nucleus of the hypothalamus disrupt the circadian rhythms of sleep and wakefulness, suggesting that neurons in these areas must relay this influence [22, 23]. Disruption of sleep-wake timing can lead to misalignment of rhythmicity in physiological variables. Sleep deprivation causes deterioration and a decrease in performance [24]. Insufficient or mistimed sleep can reduce the rhythmicity of clock-controlled genes [6, 25]. These facts suggest that the circadian clock and sleep mutually regulate each other at both the molecular and the physiological levels (Figure 1).

## Disruption of circadian clocks affects physiology and performance

Disruption of circadian rhythms may affect physical and mental health. Sleep disorders, cardiovascular disease, diabetes, obesity, cancers, inflammatory disorders, and mood disorders (depression, schizophrenia, and attention deficit) may result from dysfunction of circadian rhythms [1]. Genetic mutations and environmental desynchronization are the major causes of circadian rhythm disorders [1, 26].

Jet lag and shift work cause disruption of circadian rhythms and sleep and can cause symptoms including daytime anergia, alternating complaints of insomnia and hypersomnia, emotional disturbances, and gastrointestinal distress. Long-term shift work may lead to even more severe symptoms, such as obesity, metabolic syndromes, cardiovascular disease, and even increased cancer risk [6, 27]. Athletic performance is also compromised by disruption of the circadian clock and sleep. Athletes who travel across time zones may have transient desynchronization of their circadian timing that decreases performance [25]. These facts suggest that the circadian clock and sleep are essential for both health and behavior.

In a sleep restriction study, the reduction of sleep time to 6 hours had no overt effect on mental functioning on the first day, but the influence rose significantly with the number of days of restricted sleep [28], reflecting the effect of accruement of sleep debt. Deprivation of sleep results in a cumulative decrease in psychomotor vigilance. Interestingly, psychomotor vigilance changes in a circadian fashion [4], suggesting that both the circadian clock and sleep are important for cognitive performance. Fatigue from sleep deprivation is a significant risk factor contributing to performance decreases, and as consequence, accidents often occur at the times when people are normally asleep [29].

Exercise and performance can also feed back to influence the circadian clock (Figure 1). Independent studies have demonstrated that exercise can affect the expression, phase and periodicity of clock-associated genes [30, 31].

## Disturbances in circadian rhythms, sleep and performance in space

In space or under simulated microgravity conditions, circadian rhythms and the expression of clock-associated genes are subject to change, which leads to disruption of circadian rhythms [32–37]. In space, there are a number of factors that may influence circadian rhythms and sleep, which include microgravity, lighting conditions, the heavy workload, the shift-work schedule, confinement, and motion sickness (Figure 1). For example, in orbit, the gravitational force is very low (10^−4^-10^−6^ × *g*) [38]. In addition, the light–dark cycle is approximately 90 minutes, and astronauts are exposed to light approximately two-thirds of the time. In a space station, the lighting intensity is seriously low relative to that on Earth, although the light–dark cycle can be adjusted to be 24 hours long. Such low light intensity is below the threshold to efficiently entrain the human circadian clock [1]. In addition, the electromagnetic field and radiation exposure also greatly differ from those on the ground, and these factors might also cause changes in circadian rhythms [39, 40].

Misalignment of the circadian clock and sleep affects the nervous system, musculoskeletal system, endocrine system, and cardiovascular system, among others (Figure 1). For astronauts, circadian misalignment can increase health risks and result in a decreased ability to effectively and efficiently perform tasks [1]. Astronauts must fulfill heavy-duty mission tasks and complete spacecraft maintenance during flights. The extreme space environment, the heavy workload and the shift-work schedule can severely disrupt circadian rhythms and sleep.

Several lines of evidence suggest that a simulated or actual space environment can induce changes in circadian rhythms [36, 37, 41–44]. Independent orbital studies have demonstrated that the amplitude of body temperature rhythmicity decreases during space flight in comparison with that on the ground [45, 46]. Similarly, hypergravity leads to a dramatic decrease in body temperature and locomotor activity in rats [47]. A bed-rest experiment has been used to simulate the effects of weightlessness on the cardiovascular system and other physiological variables. Results from several bed-rest studies indicate that bed rest modifies circadian rhythms [41–44]. In certain studies, subjects on bed rest also had decreases in the amplitude of a number of physiological variables, including heart rate and blood pressure [48, 49]. Bed rest also leads to changes in the rhythms of certain hormones and electrolytes, including cortisol, melatonin, and aldosterone [50, 51]. Astronauts studied aboard the Russian Mir station had a 2-hour delay in the phase of their body temperature relative to phases on Earth [51]. Modification in the phase of certain variables, including heart rate and body temperature, has also been observed in several, but not all, studies of astronauts [52, 53]. It is noteworthy that even a small phase shift can impose a considerable influence on human performance [54]. In space, numerous interwoven environmental factors differ from those on Earth, so it is difficult to determine the effects of individual factors. Nevertheless, complementary to the space data, data from simulation studies suggest that gravitational change might be one of the causes that contribute to the changes in circadian rhythms.

In addition to circadian rhythms, disturbance in sleep is another large challenge for orbiting astronauts. A survey of the sleep patterns of space shuttle crew members demonstrated the occurrence of the most severe sleep disturbances on the first and last days of a mission, with less than 6 hours of sleep per day [53, 55]. In space, it usually takes longer for astronauts to fall asleep than that it takes on Earth [56]. An investigation of the medications used during 79 space shuttle missions indicated that 45% of those used were for the treatment of sleep disturbances [56]. A variety of sleep problems have been observed during Skylab missions, space shuttle missions and Mir missions [57]. In addition to shortening of sleep time, the sleep structure is subject to change. A reduction in the amount of slow-wave sleep and REM sleep, a shortening of REM latency and an increase in the number of arousals are typically observed during short-term space missions [49]. In contrast, the latent period for falling asleep and for the appearance of deep-sleep stages is considerably lengthened during prolonged flights. Upon return from space, sleep latency and REM latency are very short, and the percentage of REM sleep is markedly elevated, particularly during the first sleep recording after landing [53].

Sleep disturbance in space can be caused by stress, motion sickness, light flashes, emotional stress, the high work load, the abnormal work/rest schedule, thermal discomfort, noise, muscle pain, or even an unsuitable sleeping bag [58]. The consequences of sleep disturbances (insufficient sleep duration or inadequate sleep quality) may include a decline in thinking ability, alertness and judgment, as well as disorders of the immune system, which can influence the aerial work efficiency of astronauts [1, 3, 26]. Sleep deficiency also increases the risk of performance errors, which contribute to between 60% and 80% of aviation accidents [59].

Circadian and sleep deficits can result in a decrease in cognitive performance [49, 60–62]. A long-term study revealed that in astronauts in a space station, the amplitudes of oral temperature and alertness were significantly decreased, and rhythmicity was dampened [45]. Casler and Cook analyzed data from 29 studies that measured astronauts’ response time, memory, reasoning, pattern recognition, fine motor skills, and dual-task performance and revealed that several cognitive performance measures appear to be affected [47]. Similar to data from ground experiments, the orbiting astronauts show no overt changes in performance during the first days in space. However, during an 8-day mission to Mir, a decrease in fine manual control was observed [52], suggesting that the impacts of circadian and sleep disturbances on performance also have a temporal dosage effect. Dijk et al. analyzed the changes in the mood and cognitive performance of five astronauts before, during, and after 16-day or 10-day space missions, during which the sleep duration of those astronauts was only approximately 6.5 hours per day. A set of methods, including psychomotor vigilance task (PVT) measures; the Karolinska sleepiness scale (KSS); and the performance, effort, and evaluation rating scale (PEERS), revealed that there was a declining trend in performance and mood during flight [49]. In a study of crew members on the International Microgravity Laboratory (IML) space shuttle mission (STS-42) and the Canadian Astronaut Program Space Unit Life Simulation (CAPSULS) mission, a mental workload and performance experiment (MWPE) and short-term exhaustive memory and fine motor control (MEMO) experiments associated with human-computer interaction, respectively, were conducted. In these analyses, there was a significant decrease in motor performance, but not in cognitive performance [7].

In addition to natural environmental factors, certain social factors, such as confinement and isolation, may also contribute to the alterations of circadian rhythms and sleep experienced by astronauts. A significant decrease in the amplitude of wrist activity has been demonstrated in a bed-rest experiment [51]. The Mars 520-day mission demonstrated that confinement and isolation can result in inter-individually different deficits in circadian rhythms, sleep and vigilance [46]. Thus, it is critical to employ countermeasures to maintain the appropriate rhythmicity of physiology and behavior on space missions.

## Countermeasures and treatments

To prevent and alleviate the consequences of circadian and sleep deficits, countermeasures and treatments are employed to minimize the potential health and performance deterioration resulting from sleep loss and circadian rhythm disruptions. Treatments have included light therapy, administration of drug therapy, and optimization of the work-rest schedule.

In the space station, the illumination is often approximately 100 lux and usually less than 500 lux; there is not sufficient light to maintain appropriate circadian rhythms [63]. Light exposure is a commonly used approach to treat circadian misalignment and sleep disruption. For this purpose, bright light and dark goggles are used to enhance or minimize the entrainment of the circadian clock. Short-wavelength light (~460 nm to 512 nm) in the blue or green range is more effective than bright light in the adjustment of the human circadian pace [64]. Because the heavy-duty workload and the shift-based work-rest schedule also contribute to circadian and sleep disturbances, optimized scheduling helps to improve the performance and alertness of astronauts [29].

Sleep medications are also used to improve astronauts’ circadian rhythms and sleep; these medications account for nearly half of the medicines used in space stations [56]. Benzodiazepines, such as temazepam, are not ideal because they have a long onset of action and a long half-life. Instead, non-benzodiazepine hypnotics, such as zolpidem, are increasingly used. Melatonin was tested but showed no overt effect on the shuttle missions of STS-90 (Neurolab) and STS-95 [49]. Pharmacokinetic dynamics in space may differ from that those on Earth, making it necessary to assess the efficacies and side effects of medications in space. Moreover, new medications for specific use by astronauts are needed.

Mars will be the next planet for human landing. The self-rotation period of Mars is 24.65 hours, which is very close to that of Earth. Nonetheless, the light intensity on Mars is dramatically lower than that on Earth. It has been presumed that the human circadian clock will adapt to the daily day-night alternation on Mars if explorers are exposed to a higher amount of light of the appropriate spectrum. By contrast, dim lighting conditions, serving as a weak stimulus (~1.5 lux), fail to entrain the human circadian clock to the Martian day length [65].

Under moderately bright light (~450 lux), the human circadian periods of the sleep-wake cycle, core body temperature, plasma cortisol and melatonin can be entrained to either 24.65 hours or 23.5 hours [66]. During the Phoenix Mars Lander (PML) mission, approximately 87% of the subjects were entrained to the Martian day-night cycle using this light intensity [67]. This light intensity failed to entrain the human circadian clock to longer or shorter periods (e.g., 21, 27 or 28 hours) [65, 68–70], which reflects the fact that the human circadian clock can only be modifiable in a narrow window. These findings might help to acclimatize astronauts during future long-term Martian exploration.

## Perspectives

The circadian clock and sleep play critical roles in controlling physiology, cognition and performance. In space, sleep disturbance and/or circadian desynchronization have occurred during most recorded missions on which these were monitored. Both can result in decreased alertness and performance failure [1, 60, 61]. Both circadian and sleep disruptions are included in the Behavioral Health and Performance (BH&P) area as well as the Advanced Human Support Technology (AHST) area of NASA’s Bioastronautics Critical Path Roadmap [62]. During China’s Shenzhou missions, circadian rhythms and sleep have also been regarded as important areas of research emphasis by the Astronaut Center of China (ACC).

Very long-term space explorations will introduce more and more new problems that will challenge astronauts [71]. The identification of factors contributing to behavioral risks and psychiatric disorders is of great importance, allowing steps to be taken to prevent or treat these disorders [1]. For operational purposes, understanding of the fundamental mechanisms is of doubtless importance [72]. For instance, given that whether the impacts of space environmental factors on human circadian rhythms and sleep are direct or indirect remains unclear, elucidation of the mechanisms of changes in circadian rhythms and sleep is vital. Very limited in-flight studies have been conducted to evaluate circadian and sleep impacts on performance, so it will be necessary to perform systematic studies to investigate the correlation between circadian and sleep deficits and performance efficiency. In future exploration of planets other than Mars, which possesses a similar day-night cycle as Earth, modification of the robustness of the circadian clock to adapt to local light–dark conditions will be a great challenge.

## Abbreviations

SCN: Suprachiasmatic nuclei
REM: Rapid eye movement
NREM: Non-rapid eye movement
VLPO: Ventrolateral preoptic area
PVT: Psychomotor vigilance task
KSS: Karolinska sleepiness scale
PEERS: Performance evaluation and effort rating scale
BH&P: Behavioral health and performance
AHST: Advanced human support technology
IML: International micro-gravity laboratory
STS: Space shuttle mission
CAPSULS: Canadian astronaut program space unit life simulation
MWPE: Mental workload and performance experiment
MEMO: Short-term exhaustive memory and fine motor control
NASA: National aeronautics and space administration (USA)
PML: Phoenix mars lander
ACC: Astronaut centre of China.

## References

[1] Mcphee JC, Charles JB (2009). Human health and performance risks of space exploration missions: evidence reviewed by the NASA human research program.

[2] Chen S, Donald K (2014). Advances in Human Space Research - Lessons Learned and Future Directions. A Sponsored Supplement to Science: Human Performance in Space - Advancing Astronautics Research in China.

[3] Bell-Pedersen D, Cassone VM, Earnest DJ, Golden SS, Hardin PE, Thomas TL, Zoran MJ (2005). Circadian rhythms from multiple oscillators: lessons from diverse organisms. Nat Rev Genet.

[4] Van Dongen HP, Dinges DF (2005). Sleep, circadian rhythms, and psychomotor vigilance. Clin Sports Med.

[5] Mohawk JA, Green CB, Takahashi JS (2012). Central and peripheral circadian clocks in mammals. Annu Rev Neurosci.

[6] Kolla BP, Auger RR (2011). Jet lag and shift work sleep disorders: how to help reset the internal clock. Cleve Clin J Med.

[7] Newman DJ, Lathan CE (1999). Memory processes and motor control in extreme environments. IEEE Trans Syst Man Cybern C Appl Rev.

[8] Roenneberg T, Merrow M (2005). Circadian clocks - the fall and rise of physiology. Nat Rev Mol Cell Biol.

[9] Jud C, Schmutz I, Hampp G, Oster H, Albrecht U (2005). A guideline for analyzing circadian wheel-running behavior in rodents under different lighting conditions. Biol Proced Online.

[10] Wirz-Justice A, Schmid AC, Graw P, Krauchi K, Kielholz P, Poldinger W, Fisch HU, Buddeberg C (1987). Dose relationships of morning bright white light in seasonal affective disorders (SAD). Experientia.

[11] Stenberg D (2007). Neuroanatomy and neurochemistry of sleep. Cell Mol Life Sci.

[12] Steininger TL, Alam MN, Gong H, Szymusiak R, McGinty D (1999). Sleep-waking discharge of neurons in the posterior lateral hypothalamus of the albino rat. Brain Res.

[13] Fornal C, Auerbach S, Jacobs BL (1985). Activity of serotonin-containing neurons in nucleus raphe magnus in freely moving cats. Exp Neurol.

[14] Lee MG, Hassani OK, Alonso A, Jones BE (2005). Cholinergic basal forebrain neurons burst with theta during waking and paradoxical sleep. J Neurosci.

[15] Sherin JE, Elmquist JK, Torrealba F, Saper CB (1998). Innervation of histaminergic tuberomammillary neurons by GABAergic and galaninergic neurons in the ventrolateral preoptic nucleus of the rat. J Neurosci.

[16] Szymusiak R, Alam N, Steininger TL, McGinty D (1998). Sleep-waking discharge patterns of ventrolateral preoptic/anterior hypothalamic neurons in rats. Brain Res.

[17] Huang ZL, Urade Y, Hayaishi O (2011). The role of adenosine in the regulation of sleep. Curr Top Med Chem.

[18] Saper CB, Scammell TE, Lu J (2005). Hypothalamic regulation of sleep and circadian rhythms. Nature.

[19] Chou TC, Bjorkum AA, Gaus SE, Lu J, Scammell TE, Saper CB (2002). Afferents to the ventrolateral preoptic nucleus. J Neurosci.

[20] Sakurai T, Nagata R, Yamanaka A, Kawamura H, Tsujino N, Muraki Y, Kageyama H, Kunita S, Takahashi S, Goto K, Koyama Y, Shioda S, Yanagisawa M (2005). Input of orexin/hypocretin neurons revealed by a genetically encoded tracer in mice. Neuron.

[21] Watts AG, Swanson LW, Sanchez-Watts G (1987). Efferent projections of the suprachiasmatic nucleus: I. Studies using anterograde transport of Phaseolus vulgaris leucoagglutinin in the rat. J Comp Neurol.

[22] Chou TC, Scammell TE, Gooley JJ, Gaus SE, Saper CB, Lu J (2003). Critical role of dorsomedial hypothalamic nucleus in a wide range of behavioral circadian rhythms. J Neurosci.

[23] Lu J, Zhang YH, Chou TC, Gaus SE, Elmquist JK, Shiromani P, Saper CB (2001). Contrasting effects of ibotenate lesions of the paraventricular nucleus and subparaventricular zone on sleep-wake cycle and temperature regulation. J Neurosci.

[24] Lo JC, Groeger JA, Santhi N, Arbon EL, Lazar AS, Hasan S, von Schantz M, Archer SN, Dijk DJ (2012). Effects of partial and acute total sleep deprivation on performance across cognitive domains, individuals and circadian phase. PLoS One.

[25] Manfredini R, Manfredini F, Fersini C, Conconi F (1998). Circadian rhythms, athletic performance, and jet lag. Br J Sports Med.

[26] Bechtold DA, Gibbs JE, Loudon AS (2010). Circadian dysfunction in disease. Trends Pharmacol Sci.

[27] Haus EL, Smolensky MH (2013). Shift work and cancer risk: potential mechanistic roles of circadian disruption, light at night, and sleep deprivation. Sleep Med Rev.

[28] Doran SM, Van Dongen HP, Dinges DF (2001). Sustained attention performance during sleep deprivation: evidence of state instability. Arch Ital Biol.

[29] Williamson A, Lombardi DA, Folkard S, Stutts J, Courtney TK, Connor JL (2011). The link between fatigue and safety. Accid Anal Prev.

[30] Murphy BA, Wagner AL, McGlynn OF, Kharazyan F, Browne JA, Elliott JA (2014). Exercise influences circadian gene expression in equine skeletal muscle. Vet J.

[31] Okamoto A, Yamamoto T, Matsumura R, Node K, Akashi M (2013). An out-of-lab trial: a case example for the effect of intensive exercise on rhythms of human clock gene expression. J Circadian Rhythms.

[32] Baron KG, Reid KJ (2014). Circadian misalignment and health. Int Rev Psychiatry.

[33] Ferraro JS, Fuller CA, Sulzman FM (1989). The biological clock of Neurospora in a microgravity environment. Adv Space Res.

[34] Hoban-Higgins TM, Alpatov AM, Wassmer GT, Rietveld WJ, Fuller CA (2003). Gravity and light effects on the circadian clock of a desert beetle, Trigonoscelis gigas. J Insect Physiol.

[35] Mergenhagen D, Mergenhagen E (1987). The biological clock of Chlamydomonas reinhardii in space. Eur J Cell Biol.

[36] Monk TH, Buysse DJ, Billy BD, Kennedy KS, Willrich LM (1998). Sleep and circadian rhythms in four orbiting astronauts. J Biol Rhythms.

[37] Sulzman FM, Ferraro JS, Fuller CA, Moore-Ede MC, Klimovitsky V, Magedov V, Alpatov AM (1992). Thermoregulatory responses of rhesus monkeys during spaceflight. Physiol Behav.

[38] Ruyters G, Friedrich U (2006). From the Bremen Drop Tower to the international space station ISS – Ways to weightlessness in the German space life sciences program. Signal Transduct.

[39] Bliss VL, Heppner FH (1976). Circadian activity rhythm influenced by near zero magnetic field. Nature.

[40] Wever R (1970). The effects of electric fields on circadian rhythmicity in men. Life Sci Space Res.

[41] Fuller CA, Murakami DM, Sulzman FM (1989). Gravitational biology and the mammalian circadian timing system. Adv Space Res.

[42] Michikami D, Kamiya A, Fu Q, Niimi Y, Iwase S, Mano T, Suzumura A (2001). Effect of simulated microgravity exposure on thermoregulatory control of sweating. Environ Med.

[43] Mizuno K, Inoue Y, Tanaka H, Komada Y, Saito H, Mishima K, Shirakawa S (2005). Heart rate variability under acute simulated microgravity during daytime waking state and nocturnal sleep: comparison of horizontal and 6 degrees head-down bed rest. Neurosci Lett.

[44] Shiraishi M, Kamo T, Nemoto S, Narita M, Kamegai M, Baevsky RM, Funtova II (2003). Blood pressure variability during 120-day head-down bed rest in humans. Biomed Pharmacother.

[45] Monk TH, Kennedy KS, Rose LR, Linenger JM (2001). Decreased human circadian pacemaker influence after 100 days in space: a case study. Psychosom Med.

[46] Basner M, Dinges DF, Mollicone D, Ecker A, Jones CW, Hyder EC, Di Antonio A, Savelev I, Kan K, Goel N, Morukov BV, Sutton JP (2013). Mars 520-d mission simulation reveals protracted crew hypokinesis and alterations of sleep duration and timing. Proc Natl Acad Sci U S A.

[47] Casler JG, Cook JR (1999). Cognitive performance in space and analogous environments. Int J Cogn Ergon.

[48] Fischer D, Arbeille P, Shoemaker JK, O’Leary DD, Hughson RL (2007). Altered hormonal regulation and blood flow distribution with cardiovascular deconditioning after short-duration head down bed rest. J Appl Physiol (1985).

[49] Dijk DJ, Neri DF, Wyatt JK, Ronda JM, Riel E, Ritz-De Cecco A, Hughes RJ, Elliott AR, Prisk GK, West JB, Czeisler CA (2001). Sleep, performance, circadian rhythms, and light–dark cycles during two space shuttle flights. Am J Physiol Regul Integr Comp Physiol.

[50] Liang X, Zhang L, Wan Y, Yu X, Guo Y, Chen X, Tan C, Huang T, Shen H, Chen X, Li H, Lv K, Sun F, Chen S, Guo J (2012). Changes in the diurnal rhythms during a 45-day head-down bed rest. PLoS One.

[51] Liang X, Zhang L, Shen H, Chen X, Wan Y, Li L, Liang L, Yu X, Guo Y, Yu J, Shu W, Tan C, Lv K, Xiao Y, Chen X, Chen S, Guo J (2014). Effects of a 45-day head-down bed rest on the diurnal rhythms of activity, sleep and heart rate. Biol Rhythm Res.

[52] Manzey D, Lorenz B (1998). Mental performance during short-term and long-term spaceflight. Brain Res Brain Res Rev.

[53] Czeisler CA, Gooley JJ (2007). Sleep and circadian rhythms in humans. Cold Spring Harb Symp Quant Biol.

[54] Burgess HJ, Legasto CS, Fogg LF, Smith MR (2013). Can small shifts in circadian phase affect performance?. Appl Ergon.

[55] Santy PA, Kapanka H, Davis JR, Stewart DF (1988). Analysis of sleep on Shuttle missions. Aviat Space Environ Med.

[56] Putcha LBK, Marshburn TH, Ortega HJ, Billica RD (1990). Pharmaceutical use by U.S. astronauts on space shuttle missions. Aviat Space Environ Med.

[57] Barger LK, Flynn-Evans EE, Kubey A, Walsh L, Ronda JM, Wang W, Wright KP, Czeisler CA (2014). Prevalence of sleep deficiency and use of hypnotic drugs in astronauts before, during, and after spaceflight: an observational study. Lancet Neurol.

[58] Gundel A, Polyakov VV, Zulley J (1997). The alteration of human sleep and circadian rhythms during spaceflight. J Sleep Res.

[59] Weigmann DA, Shappell SA (2003). A Human Error Approach to Aviation Accident Analysis: the Human Factors Analysis and Classifi Cation System.

[60] Holley DC, DeRoshia CW, Moran MM, Wade CE (2003). Chronic centrifugation (hypergravity) disrupts the circadian system of the rat. J Appl Physiol (1985).

[61] Benke T, Koserenko O, Watson NV, Gerstenbrand F (1993). Space and cognition: the measurement of behavioral functions during a 6-day space mission. Aviat Space Environ Med.

[62] Mallis MM, DeRoshia CW (2005). Circadian rhythms, sleep, and performance in space. Aviat Space Environ Med.

[63] West KE, Jablonski MR, Warfield B, Cecil KS, James M, Ayers MA, Maida J, Bowen C, Sliney DH, Rollag MD, Hanifin JP, Brainard GC (2011). Blue light from light-emitting diodes elicits a dose-dependent suppression of melatonin in humans. J Appl Physiol (1985).

[64] Fucci RL, Gardner J, Hanifin JP, Jasser S, Byrne B, Gerner E, Rollag M, Brainard GC (2005). Toward optimizing lighting as a countermeasure to sleep and circadian disruption in space flight. Acta Astronaut.

[65] Wright KP, Hughes RJ, Kronauer RE, Dijk DJ, Czeisler CA (2001). Intrinsic near-24-h pacemaker period determines limits of circadian entrainment to a weak synchronizer in humans. Proc Natl Acad Sci U S A.

[66] Scheer FA, Wright KP, Kronauer RE, Czeisler CA (2007). Plasticity of the intrinsic period of the human circadian timing system. PLoS One.

[67] Barger LK, Sullivan JP, Vincent AS, Fiedler ER, McKenna LM, Flynn-Evans EE, Gilliland K, Sipes WE, Smith PH, Brainard GC, Lockley SW (2012). Learning to live on a Mars day: fatigue countermeasures during the Phoenix Mars Lander mission. Sleep.

[68] Czeisler CA, Duffy JF, Shanahan TL, Brown EN, Mitchell JF, Rimmer DW, Ronda JM, Silva EJ, Allan JS, Emens JS, Dijk DJ, Kronauer RE (1999). Stability, precision, and near-24-hour period of the human circadian pacemaker. Science.

[69] Aschoff J (1965). Circadian Rhythms in Man a self-sustained oscillator with an inherent frequency underlies human 24-hour periodicity. Science.

[70] Lewis PR, Lobban MC (1957). The effects of prolonged periods of life on abnormal time routines upon excretory rhythms in human subjects. Q J Exp Physiol Cogn Med Sci.

[71] Sandal GM (2001). Psychosocial issues in space: future challenges. Gravit Space Biol Bull.

[72] Wang D, Zhang L, Liang X, Shen H, Chen X, Wan Y, Guo J, Donald K (2014). Space Meets Time: Impact of Gravity on Circadian/Diurnal Rhythms. A Sponsored Supplement to Science: Human Performance in Space - Advancing Astronautics Research in China.

